# A Questionnaire on Relative Deprivation of University Students and Its Application in Measuring Mental Health

**DOI:** 10.3389/fpsyg.2022.832927

**Published:** 2022-02-24

**Authors:** Liuzhan Jia

**Affiliations:** Management School, Henan University of Technology, Zhengzhou, China

**Keywords:** relative deprivation, social comparison, college students, psychology healthy, cognitive evaluation, emotional experience

## Abstract

**Objective:**

Relative deprivation is associated with collective and individual variables in psychology. However, so far, there are few studies on measuring the relative deprivation of university students. Therefore, this study designs the University Students’ Relative Deprivation Questionnaire (USRDQ), verifies its validity and reliability, and then uses it to measure the mental health of students.

**Methods:**

After reviewing the relevant studies and conducting a theoretical analysis and an open questionnaire survey, this article determined the structural dimension of USRDQ. A total of 103 university students were selected to take the open questionnaire survey, 200 were selected to engage in the item analysis, exploratory factor analysis, and internal consistency reliability test, 257 were selected to engage in the confirmatory factor analysis, and 287 were selected to take the retest reliability.

**Results:**

The USRDQ includes 19 items under the three dimensions, namely, social comparison, cognitive evaluation, and emotional experience. Factor loads range from 0.49 to 0.87, which accounted for 63.39% of the total variation. The questionnaire has good fitting indicators (χ^2^/*df* = 2.64, NFI = 0.89, CFI = 0.93, GFI = 0.91, RMSEA = 0.08). The Cronbach’s α coefficient of the questionnaire is 0.916, and the coefficients of the three factors range from 0.805 to 0.934. The results of the survey show that the relative deprivation of students is quite high with a mean of 76.78 and a standard deviation of 16.96.

## Introduction

### Origin and Development of Relative Deprivation

Relative deprivation refers to a cognitive evaluation of an individual on his or her disadvantageous situation and the resultant feelings of anger and resentment after making a social comparison with others (Smith et al., 2012). This concept was first put forward by Stouffer et al. (1949) to evaluate the dissatisfaction of US soldiers during World War II. Gurr (1970) believed that relative deprivation was the discrepancy of value expectations and value capability, the gap of which would be explained by political violence. Displeasure arises when people feel unfairly deprived of some resources that people similar to them have but they do not (Crosby, 1976). Relative deprivation can also be generated when one makes a social comparison with the history of an individual, other people in the same group, or members of other groups (Walker and Pettigrew, 1984).

Relative deprivation can be divided into relative deprivation of an individual (referred to as individuals) and relative deprivation of a group (referred to as groups). Relative deprivation has major impacts on many consequence factors, such as group behavior, individual achievement and deviant behavior, intergroup attitudes, intergroup discrimination, aggressive behavior, prosocial tendencies (Kassab et al., 2021; Xiong et al., 2021), national identification, mental health, physical health, psychological stress, biochemical disorder, and suicide risk (Mishra and Meadows, 2018; Leviston et al., 2020). Korotayev and Shishkina (2019) equaled relative deprivation to the decrease of subjective feeling of happiness in their study, and it was considered a powerful indicator for predicting destabilization.

### Relative Deprivation Dimensions

Relative deprivation has three dimensions, namely, social comparison, cognitive evaluation, and emotional experience (Smith et al., 2012). Social comparison refers to the comparison of an individual with oneself, people in his/her own group, and people in other groups in different time dimensions. Social comparison is a prerequisite condition for relative deprivation. If there is no social comparison, relative deprivation may never occur. The second is cognitive evaluation, which refers to the perception of an individual on disadvantageous situation derived from social comparison. People believe that their disadvantageous situation is unfair and they deserve better treatment. The third is emotional experience. An individual in a disadvantageous situation will bear anger and resentment. This is also a key component of relative deprivation. Relative deprivation is the result of a cognitive evaluation of an individual on emotional experience from unfair treatment.

### Relative Deprivation Measurement

Researchers have paid much attention to relative deprivations influencing factors and consequence variables and have achieved productive results. However, studies on measuring tools of relative deprivation are not adequate. At present, there are mainly three ways to measure relative deprivation. The first way uses statistical indicators in the socioeconomic field to measure relative deprivation, such as the Gini coefficient (Silber and Verme, 2010) and the Yitzhaki Index (based on the income level) (Adjaye-Gbewonyo and Kawachi, 2012; Kuo and Chiang, 2013). The Yitzhaki index is the generalization of one parameter, mainly depending on the gap between the past and the present income distribution, rather than income distribution (Bossert and D’Ambrosio, 2020). This method applies various objective statistical indicators in economics to reflect the level of relative deprivation by describing the income gap.

The second way uses social comparison results to reflect the level of relative deprivation. For example, in the study of Ma (2012), social comparison was made in one or more aspects, and the gap of the comparison was used to measure relative deprivation. The aspects of social comparison include working condition, wealth, interpersonal relationship, housing condition, and the quality of living environment (Abrams and Gran, 2012; Koschate et al., 2012). The gap between the socioeconomic status of a family and a specific reference object was used to measure relative deprivation (Zhang and Tao, 2013). Although this method took social comparison into consideration when measuring relative deprivation, it ignored emotional experience. Therefore, it had limitations as it only addressed specific aspects of social comparison.

The third way develops a relative deprivation scale. Using the Individual Relative Deprivation Scale (IRDS), Zoogah (2010) focused on social comparison in terms of income. IRDS used six items to measure cognitive and affective relative deprivation. For example, several of these items could be “Foreign-educated employee were paid more” and “I feel resent my payment was less than foreign-educated employees.” However, these items were small in number and lacked variety, mainly focusing on payment but ignoring other aspects. The Personal Relative Deprivation Scale (PRDS) was used in the study of Callan et al. (2011) to measure the perception of relative deprivation and the derived dissatisfaction and resentment. But, in his study, neither enough items were included in the scale, nor structural dimensions were classified and verified. Wickham et al. (2013) designed the Perceived Relative Deprivation Scale in Childhood (PRDSC), which divided the relative deprivation into two dimensions, namely, perceived relative deprivation (12 items) and family social capital (4 items). However, it was inconsistent with the basic concept of relative deprivation. Besides, the psychometric indicators of this scale presented some weaknesses. The exploratory factor analysis showed that this scale contained two dimensions, namely, perceived relative deprivation and family social capital, but the confirmatory factor analysis (CFA) indicated that there were three dimensions, namely, neighborhood perceived relative deprivation, global perceived relative deprivation, and perceived family social capital. And the CFA analysis had set some residual error to be relevant. Besides, the structural validity was far from being satisfying. This scale mainly targeted at the perception of relative deprivation in childhood with a focus on social comparison of material life. Therefore, its application was limited.

Although there were numerous indicators to measure relative deprivation, few studies probed into how the measurement reflected the essence of mind for relative deprivation, and empirical data to verify the study were lacking (Hounkpatin et al., 2020).

### Relative Deprivation and Mental Health of University Students

Relative deprivation is associated with poor mental health. One study proved that perceived relative deprivation in childhood was likely to link with sub-syndrome psychotic symptoms (Wickham et al., 2014). Researchers found the relationship between relative deprivation and mental health of university students. The study by Smith and Ryan found (Smith et al., 2020) that relative deprivation caused increased anxiety and depression of an individual, the negative effect of which might last for years. The past study focused on relative deprivation of an individual but neglected the effect of sentiment relative deprivation on the mental health of an individual. University students were peers coming from different backgrounds. Studies on their relative deprivation in childhood alone were not enough to account for various aspects of relative deprivation. Therefore, this study aimed at developing a more comprehensive relative deprivation scale to measure relative deprivation.

### Purpose of the Study

The analysis on relative deprivation measurement showed that previous scales were less reliable and valid, and failed to meet the criteria of psychometrics according to the “Standards for educational and psychological testing” (American Educational Research Association et al., 2014). Given that there is no scientifically effective relative deprivation questionnaire targeting university students in China, this study, based on the basic definition and interpretation of relative deprivation, designed a relative deprivation questionnaire for Chinese university students.

Based on the abovementioned conceptual analysis, this article agrees that relative deprivation should include three dimensions. The first dimension is social comparison, in which an individual compares his/her current situation with that in the past, that of others in his/her group, and that of others in other groups. The second dimension is cognitive evaluation, in which an individual believes that his/her disadvantageous situation is unfair after making a social comparison. The third dimension is emotional experience, in which after an individual finds out that he/she is in a disadvantageous situation, he/she will feel anger, resentful, and unbalanced.

## Subjects and Methods

### Subjects

Sample 1: for an open questionnaire survey, the survey was carried out in universities in Wuhan. University students and postgraduates were selected on a class basis. A total of 118 questionnaires were sent out, and 103 valid questionnaires were collected, including 46 from male students and 57 from female students; 47 were from juniors, 39 from seniors, and 17 from postgraduates; and these students aged from 21 to 28 years, with an average age of (22 ± 3) years.

Sample 2: for the analysis of questionnaire items, reliability analysis, and exploratory factor analysis, the survey was carried out in universities in Wuhan, Tianjin, and Chongqing. University students and postgraduates were selected on a class basis. A total of 230 questionnaires were sent out, and 200 valid questionnaires were collected, including 78 from male students, 111 from female students, and 9 from without identified gender; 2 were from freshmen, 70 from sophomores, 69 from juniors, 26 from seniors, 24 from postgraduates, and 9 from without identified grade; and these students aged from 20 to 26 years, with an average age of (22 ± 2) years.

Sample 3: for CFA and analysis of the questionnaire score, the survey was carried out in universities in Wuhan, Chongqing, and Zhengzhou. University students and postgraduates were selected on a class basis. A total of 300 questionnaires were sent out, and 257 valid questionnaires were collected, including 96 from male students, 144 from female students, and 17 from without identified gender; 27 were from freshmen, 81 from sophomores, 74 from juniors, 28 from seniors, 30 from postgraduates, and 17 from without identified grades; and these students aged from 19 to 26 years, with an average age of (22 ± 3) years.

Sample 4: for the retest of questionnaire reliability, the survey was carried out in universities in Zhengzhou, Wuhan, and Chongqing. Graduate students and postgraduates were selected on a class basis. A total of 300 questionnaires were sent out, and 287 questionnaires were collected, including 124 from male students and 163 from female students; 32 were from freshmen, 90 from sophomores, 88 from juniors, 30 from seniors, and 47 from postgraduates; these students aged from 19 to 27 years, with an average age of (23 ± 3) years. The respondents of the survey are shown in Table 1. The sample size was applied to basic power analysis according to the general criteria.

**TABLE 1:** Respondents of the survey.

Sample no.		1	2	3	4
Gender	Male	46	78	96	124
	Female	57	111	144	163
	Unlabeled	0	9	17	0
Grade	Freshmen	47	2	27	32
	Sophomores	0	70	81	90
	Juniors	39	69	74	88
	Seniors	0	26	28	30
	Postgraduates	17	24	30	47
	Unlabeled		9	17	0
Average age		22 ± 3	22 ± 2	22 ± 3	23 ± 3
Sum		103	200	257	287

### Methods

#### Open Questionnaire Survey

An open questionnaire survey was conducted among the abovementioned students. The survey focused on the perception of an individual on relative deprivation, including how to compare oneself with others, the perceived situation after comparison, the feelings after comparison, how to treat the comparison results, and the fairness of situation of an individual. A total of 118 questionnaires were sent out, of which 103 valid questionnaires were collected. The remaining 15 copies were invalid. The research conducted a statistical analysis to the item frequency and classified those with high frequencies. The frequency was between 11 and 103. Items that fell into the category of social comparison were as follows: after being compared with different reference objects, an individual found himself/herself in a disadvantageous situation (frequency 103) and an individual made less achievements compared with those having similar abilities (frequency 103). Items that fell into the category of cognitive evaluation were as follows: an individual thought it was unfair for himself/herself to be inferior to others (frequency 51), and under the same conditions, an individual should be treated the same as others (frequency 11). Items that fell into the category of emotional experience were as follows: an individual felt angry (frequency 34), resentful, and frustrated after finding himself/herself inferior to others (frequency 31).

#### Collecting Items and Designing the Initial Questionnaire

First, by referring to previous questionnaires (Zoogah, 2010; Callan et al., 2011), the research selected two frequently used items, namely, “I feel resentful because of my inferior condition” and “I feel unsatisfied after comparing with others.” The research translated and back-translated these two items. Then, after sorting and analyzing the items of the open questionnaire, the research invited doctor candidates in social psychology and postgraduates in senior grade to evaluate whether these items were professional, selected 23 items, and described them in a scientific and concise manner. Finally, through expert evaluation and small-sample pretest, the research designed the initial University Students’ Relative Deprivation Questionnaire (USRDQ) containing 25 items, and each item was randomly placed in the questionnaire. Among them, nine items fell into the category of social comparison, including social comparisons between an individual and different reference objects. Eight items fell into the category of cognitive evaluation, including the attitudes of an individual toward fairness after making social comparison. Eight items fell into the category of emotional experience, including the negative emotional experience of an individual after making social comparison. A 7-point Likert-type scale was applied to this survey, with 7 for completely agree, 6 for totally agree, 5 for mostly agree, 4 for slightly agree, 3 for slightly disagree, 2 for mostly disagree, and 1 for completely disagree.

### Statistical Methods

SPSS 21.0 was used for the item analysis, exploratory factor analysis, and reliability analysis, and AMOS 20.0 was used for the CFA. This study chose items that could differentiate low- and high-score groups using item analysis, and extract principal dimensions of USRDQ using the exploratory factor analysis. AMOS 20.0 was used to test, verify, and compare the structural dimensions of USRDQ through the exploratory factor analysis.

## Results

### Pilot Study

First, the item analysis was conducted to the pretest questionnaire. Scores of each respondent were added, and items were arranged from that with the highest score to the one with the lowest score. The first 27% of the items were considered in the high-score group, and the latter 27% were considered in the low-score group. A verification test of these items was conducted. The *t*-test finds that each item of the questionnaire is highly different from one another, the decision value is greater than 3, and the significance is *p* < 0.001. Second, an item-score correlation test was carried out. Results show that the correlation between each item and the total score of the questionnaire is relatively high, the correlation coefficients are from 0.42 to 0.76, and the significance is *p* < 0.01.

### Questionnaire Scores

The average score of the relative deprivation questionnaire is (96.31 ± 21.42). The average score for social comparison is (34.23 ± 8.31), that for cognitive evaluation is (30.71 ± 7.34), and that for emotional experience is (31.53 ± 7.31).

### Structure Validity

#### The Exploratory Factor Analysis

The exploratory factor analysis was used to make descriptive statistics of the survey. The analysis results are shown in Table 2. There are differences between items in high- and low-score groups, and the significance of an independent sample test is *p* < 0.001.

**TABLE 2:** Descriptive analysis of the survey.

Item no.	Mean	*SD*	Skewness	Kurtosis
1	3.97	1.433	–0.17	–0.03
2	3.69	1.21	0.14	0.59
3	5.04	1.60	–0.50	–0.32
4	4.35	1.44	0.08	–0.12
5	3.73	1.32	0.01	0.16
6	4.53	1.46	–0.01	–0.15
7	4.03	1.56	0.03	–0.48
8	3.48	1.38	0.24	–0.08
9	5.12	1.64	–0.59	–0.24
10	4.42	1.37	0.08	0.07
11	3.72	1.27	–0.08	0.02
12	4.34	1.51	0.09	–0.53
13	4.07	1.51	–0.16	–0.23
14	3.74	1.31	0.14	0.10
15	4.71	1.51	–0.09	–0.44
16	3.93	1.26	0.14	0.56
17	3.65	1.29	–0.09	0.47
18	3.56	1.43	0.10	–0.12
19	3.77	1.46	0.13	–0.14
20	3.36	1.17	0.11	0.74
21	5.17	1.57	–0.55	–0.48
22	4.22	1.37	0.31	–0.32
23	3.34	1.18	0.18	0.73
24	4.18	1.55	0.16	–0.28
25	4.15	1.42	–0.10	–0.07

First, the Bartlett’s sphere test and KMO test were conducted. In the Bartlett’s sphere test, χ^2^ = 2,256.90 (*df* = 171, *p* < 0.001). KMO value is 0.89. These results indicate that the overall correlation matrix has common factors and is suitable for the factor analysis.

The principal component analysis method was used to extract factors. The factors with eigenvalues greater than 1 were selected. The orthogonal maximum variance method for rotation factor was set, and the factor load criteria greater than 0.4 were used in the exploratory factor analysis. As a result, the common degree value of each item ranges from 0.36 to 0.89. The gravel chart shows that, starting from the fourth factor, the curve gradually becomes flat, indicating that it is appropriate to extract the three factors.

According to the suggestions by Wu (2010), some items were excluded. Three items that had double loads in the rotated component matrix were deleted. And three other items that were difficult to fall into any category were deleted. The final questionnaire retained 19 items, including three factors with an eigenvalue greater than 1. The rotated factor matrix is shown in Table 3. The explained variance of the three factors is 25.28%, 21.19%, and 16.89%, respectively, and the total explained variance is 63.39%.

**TABLE 3:** Factor load of each item of the questionnaire.

Factor 1: Social comparison		Factor 2: Cognitive evaluation		Factor 3: Emotional experience	
**Item**	**Load**	**Item**	**Load**	**Item**	**Load**
1	0.85	2	0.80	3	0.87
7	0.81	8	0.76	9	0.73
13	0.79	14	0.75	15	0.75
19	0.76	20	0.75	21	0.58
25	0.76	23	0.73	22	0.49
24	0.75	17	0.72		
18	0.74	11	0.51		
Sum	25.28%		21.19%		16.89%

The three factors are shown in Table 3. Factor 1 is a social comparison, which contains 7 items. For example, an individual finds himself/herself in a disadvantageous situation after making a social comparison with his/her past situation, others from the same group, or others from other groups. Factor 2 is a cognitive evaluation, which contains 7 items. For example, an individual finds it unfair that he/she is inferior to others. Factor 3 is an emotional experience, which contains 5 items. For example, an individual feels angry and furious after making a social comparison to others. The correlation analysis shows that the score of social comparison is positively correlated with those of cognitive evaluation scores and emotional experience (*r* = 0.49 and 0.55, *p* < 0.001), the score of cognitive evaluation is positively correlated with those of emotional experience (*r* = 0.28, *p* < 0.001), and the score of all these three dimensions is positively correlated with the total score of the questionnaire (*r* = 0.84, 0.73, and 0.74, and all *p-*values < 0.001).

#### Confirmatory Factor Analysis

**FIGURE 1:**
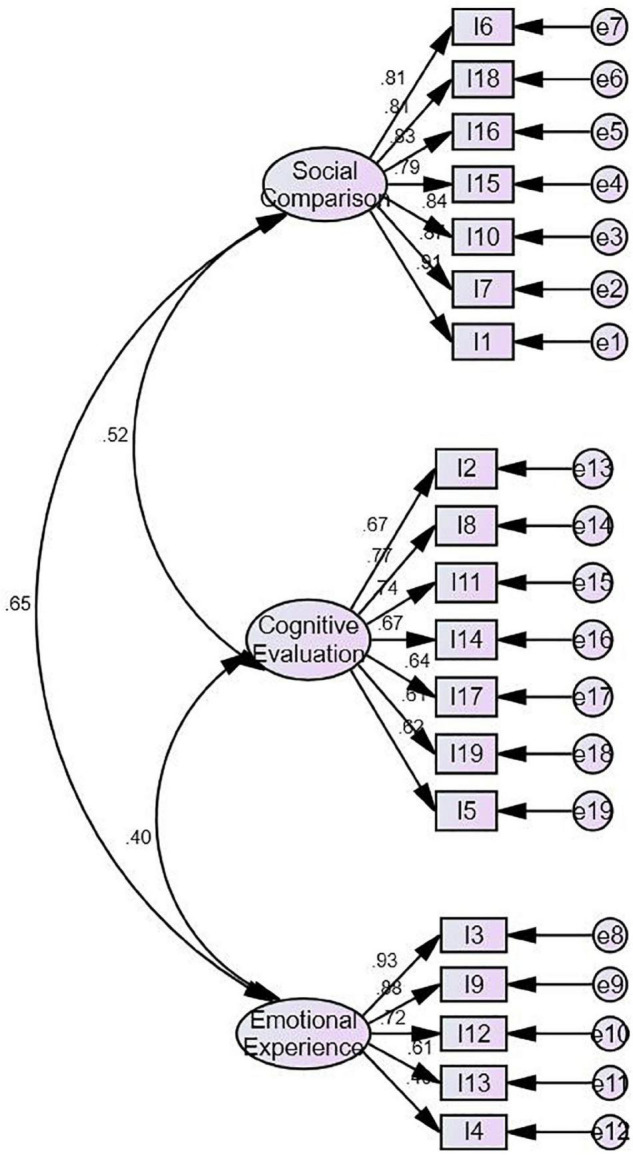
The oval represents the unobserved variable, the square represents the items of the unobserved variable to be measured, and the small circle represents the error.

This study used AMOS 20.0, a maximum likelihood method to conduct the CFA on the questionnaire. Missing data were filled using the series mean method. Results show a good fitting of each index in Table 4. The operation results and standardized pathway coefficient are shown in Figure 1.

**TABLE 4:** Comparison of compete models.

Model	Fitness index									
	χ^2^	*df*	χ^2^*/df*	NFI	RFI	IFI	TLI	CFI	GFI	RMSEA
Single-factor model	1100.49	152	7.24	0.66	0.62	0.69	0.65	0.69	0.62	0.16
Two-factor model	1098.975	151	7.28	0.66	0.61	0.69	0.65	0.69	0.63	0.16
Supposed model	391.29	149	2.64	0.89	0.86	0.92	0.91	0.93	0.91	0.08
										

Then, the study compared the compete models using the CFA. The compete model consists of a single factor model and a two-factor model. AMOS 20.0 was used for model comparison, and the fitness index is summarized in Table 4. In the single factor model, all items were taken as one comprehensive dimension of relative deprivation. In the two-factor model, social comparison and cognitive evaluation were combined as one factor, since they both belonged to social cognition and were rational intellectual activities. Emotional experience was the other factor, since it was an emotional activity.

According to the contrast model, this study believed that the three-factor model was the best one for theory hypothesis and data analysis.

### Content Validity Analysis

Content validity refers to the extent to which the items on a test are fairly representative of the entire domain that the test seeks to ensure. Items in this questionnaire were identified based on related theories and the open questionnaire survey and were finally determined after discussions with psychologists and PhDs in social psychology. This study intended to ensure that USRDQ interpreted relative deprivation correctly and addressed the features of university students. When designing the questionnaire, the description, details, semantics, and other aspects of the items were evaluated repeatedly in order to ensure that the questionnaire had high content validity. As relative deprivation is an important social variable, it can be used to predict lots of significant consequence variables, such as collective action, individual achievement, individual deviance, intergroup attitudes, and mental health (Smith et al., 2012).

### Reliability Analysis

Internal consistency reliability: the internal reliability for the entire USRDQ is 0.916, and the internal consistency reliabilities of social comparison, cognitive evaluation, and emotional experience are 0.934, 0.869, and 0.805, respectively.

Retest reliability: the retest reliability for the entire USRDQ is 0.919, and the retest reliabilities of social comparison, cognitive evaluation, and emotional experience are 0.932, 0.853, and 0815, respectively. The reliability of the relative deprivation and its dimension is shown in Table 5.

**TABLE 5:** Reliability coefficient of relative deprivation.

	Internal test reliability	Re-test reliability
Overall questionnaire	0.916	0.919
Social comparison	0.934	0.932
Cognitive evaluation	0.869	0.853
Emotional experience	0.805	0.815

The reliability analysis and validity confirm analysis prove that USRDQ has satisfying reliability and validity. According to the suggestions by Wu (2010) and based on acknowledged standards, the reliability and retest reliability are all higher than 0.8, which meets the requirement, and the fitness index of CFA also meets the requirement. The items of USRDQ are listed in Table 6.

**TABLE 6:** University students’ relative deprivation questionnaire (partly).

Factor	Item
Social comparison	After comparing with his/her previous situation, the individual finds himself/herself in a worse situation.
	After comparing with his/her ideal situation in future, the individual finds himself/herself be far from an ideal situation.
	After comparing with others in his/her group, the individual finds him/her inferior to others.
Cognitive evaluation	It is unfair that an individual is in a worse situation than before.
	It is unfair that an individual is far from being in an ideal situation.
	It is unfair that an individual is inferior to others in his/her group.
Emotional experience	An individual feels angry after making social comparison.
	An individual feels furious after making social comparison.

## Relative Deprivation Status of University Students

From the analysis, it can be found that university students have a high level of relative deprivation, with an average score of 76.78 and a standard deviation of 19.69. The average score of social comparison is 27.82, and the standard deviation is 8.65. The average score of cognitive evaluation is 25.21, and the standard deviation is 6.18. The average score of emotional experience is 24.06, and the standard deviation is 5.82. The general results of the survey are shown in Table 7.

**TABLE 7:** The relative deprivation status.

	Average score	Standard deviation
Overall questionnaire	76.78	16.96
Social comparison	27.82	8.65
Cognitive evaluation	25.21	6.18
Emotional experience	24.06	5.82

The gender difference test was applied to the total score of the questionnaire and the score of each dimension, and results show that there is no significant difference between men and women in the overall relative deprivation and each dimension.

**FIGURE 2:**
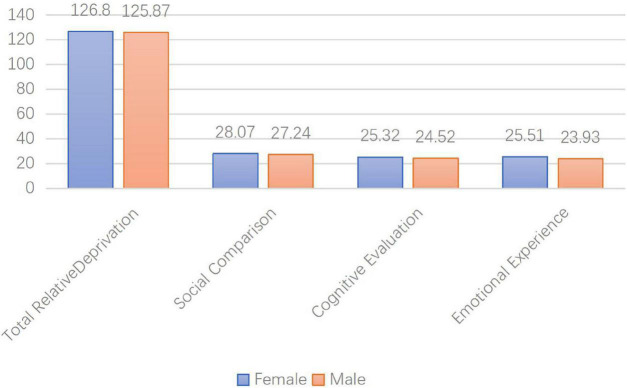
The lateral axis is the overall relative deprivation and three dimensions; the vertical axis is the value.

For the overall relative deprivation, *t* = 0.248 (*p* = 0.805); for social comparison, *t* = 0.64 (*p* = 0.523); for cognitive evaluation, *t* = 0.806 (*p* = 0.421); for emotional experience, *t* = 0.661 (*p* = 0.510). Gender differences are shown in Figure 2.

The ANOVA analysis of grade has found that grade has a significant difference in relative deprivation, *F* (4, 235) = 54.79, *p* < 0.01. The analysis of ANOVA reveals that relative deprivation level increases with the grade. The ANOVA is summarized in Table 8.

**TABLE 8:** The ANOVA of grade difference.

		Grade				
Relative deprivation	Freshmen (*n* = 27)	Sophomores (*n* = 81)	Juniors (*n* = 74)	Seniors (*n* = 28)	Postgraduates (*n* = 30)	*F*(4, 235)
Mean	53.55	69.56	77.52	86.93	90.87	
*SD*	12.30	11.54	11.39	10.04	8.22	54.79**

**p < 0.01.

Then, the research continued the *post-hoc* test using the LSD methods, and the multiple comparisons were used to identify the difference in grades. The relative deprivation level of sophomores and freshmen exists a significant difference (*M* = 16.05, *p* < 0.01). The relative deprivation level of juniors and sophomores exists a significant difference (*M* = 7.93, *p* < 0.01). The relative deprivation level of seniors and juniors exists a significant difference (*M* = 9.41, *p* < 0.01). Finally, the relative deprivation level of postgraduates and seniors exists no significant difference (*M* = 3.93, *p* = 0.18).

## Discussion

### The Structural Dimension Analysis on University Students’ Relative Deprivation Questionnaire

Stouffer et al. (1949) proposed the concept of relative deprivation, but they did not further explore the methods to measure relative deprivation, rather they used it as an explanation of a certain social phenomenon. This left room for studying how to measure relative deprivation. Walker and Pettigrew (1984) reviewed relative deprivation and put forward several relevant issues, such as the difference between egoistic and fraternalistic, the measurement level, the difference between cognitive and affective, the difference between absolute and relative, and specification on the reference object and on comparative dimensions. Previously, researchers paid less attention to measuring relative deprivation, which might be because it was a concept originated from sociology. They placed a greater emphasis on the use of socioeconomic statistical indicators, rather than measuring relative deprivation from a psychological perspective.

In this study, after reviewing the literature and conducting an open questionnaire survey, reliability analysis, and validity analysis, the research designed the USRDQ, which included three dimensions, namely, social comparison, cognitive evaluation, and emotional experience. Respondents were required to make social comparison with different reference objects, evaluate the results after comparisons, and find out how they feel after evaluations. The USRDQ addressed the six questions proposed by Walker and Pettigrew (1984). It differentiated egoistic and fraternalistic, cognitive and affective, and absolute and relative, and drew a conclusion on the dimensions.

The three dimensions of relative deprivation are closely related to each other, and they are complementary and mutually reinforcing. Social comparison is a necessary condition for relative deprivation. In social comparison, an individual compares his/her own situation with various reference objects. After making a social comparison, an individual will inevitably evaluate the comparison results, that is, what kind of status and situation he/she is compared with reference objects. Cognitive evaluation results in emotional experience. If an individual finds himself/herself inferior to others in certain aspects and believes that the disadvantageous situations are unfair and due to reasons other than himself/herself, he/she will produce anger and resentment. The individual will feel resentful about the social system and believes that it results in his/her disadvantageous situation. Resentment gives rise to the judgment of people about fairness. It is a temporary moral emotion with a clear focus, that is, the unfair institutional mechanism (Mackie et al., 2000). Anger is the result of unfair treatment, and it would generate aggression against others (Grant, 2008).

This questionnaire designed not only meets the requirements of psychometrics but also fits the basic concept of relative deprivation. Previous studies emphasized that relative deprivation included cognitive component and emotional component. This study found that among the three dimensions of relative deprivation, social comparison, and cognitive evaluation belonged to the cognitive component, and emotional experience belonged to the emotional component. This study is consistent with the theory of relative deprivation. Social comparison is a necessary component of relative deprivation. After making a social comparison, an individual will ask about his/her own situation. This is the cognitive component of relative deprivation. Please note that the cognitive component is not always true, as it is likely to be influenced by prejudice. When an individual finds that he/she is inferior to others in a certain aspect and believes that such disadvantageous situation is unfair, anger and resentment will occur.

Relative deprivation is divided into relative deprivation of an individual and relative deprivation of a group. Runciman (1966) and Smith and Pettigrew (2015) thought that relative deprivation referred to an individual or a group was deprived of certain aspects compared to similar people or groups. In addition, Grant et al. (2015) distinguished the concept of relative deprivation of an individual and that of a group. The questionnaire of this study addresses both, so that the measurement of relative deprivation is more comprehensive.

### The Comparison of University Students’ Relative Deprivation Questionnaire With Other Scales

Compared to previous relative deprivation questionnaires, the one designed in this study is more in line with the nature of relative deprivation and meets the criteria of psychometrics. The previous method using socioeconomic statistical indicators to reflect the relative deprivation lacked reliability and validity, as it ignored internal psychological feelings. However, USRDQ considers the nature of relative deprivation as a psychological feeling and it is more appropriate to measure cognitive evaluation and emotional experience produced by social comparisons.

The previous method focused on the characteristics of social comparison but paid little attention to emotional experience. The research thought it was incomplete that neglect the feeling aspect of relative deprivation, USRDQ measures social comparison, cognitive evaluation, and emotional experience of relative deprivation. The measurement is multifaceted. Compared with the relative deprivation questionnaire in previous studies, USRDQ includes the aspects of different social comparisons and has a wide range of applications. In terms of context and structural dimensions, USRDQ is superior to the PRDS (Callan et al., 2011). In terms of the differentiation and verification of structural dimensions, USRDQ is better than the PRDSC (Wickham et al., 2013). The USRDQ designed in this study has made much improvement based on social and economic indicators or social comparison results, which is conductive to measuring relative deprivation in a scientific way.

The factor dimension obtained through the exploratory factor analysis of this study is basically the same as the theoretically constructed structure, which is a three-dimensional structure that includes social comparison, cognitive evaluation, and emotional experience. Items of this questionnaire are based on relevant scales and theories (Zoogah, 2010; Wickham et al., 2013). The correlation analysis of the three dimensions of relative deprivation, the score of each dimension, and the total score indicate that the three dimensions are independent and different from each other. Through the CFA, it is found that the fitting indicators of USRDQ are relatively good, and each item in the questionnaire has a higher factor load on latent variables.

### Relative Deprivation in the Current Society

At present, many people feel that they are in a state of being deprived, and they have not achieved what they deserve. The relative deprivation level is quite high. Groups with low social status and income present a high level of relative deprivation. Even government officials, white-collar workers, and intellectuals are trapped in the same situation. Relative deprivation reflects how people react to social changes in their circumstances. This phenomenon may be related to the selection of comparison objects and the way to make social comparison. In a stable social order and under stable social norms, people usually choose to compare with those who are similar to them in ability or in certain respects. However, the society is changing thoroughly. People may not necessarily compare with those who have the homogeneity, but with those who are different from them, such as celebrities, high-ranking government officials, and wealthy businessmen, finding only that they are in a disadvantageous situation. This is because people tend to compare with those who are superior to them. Therefore, this study believes that, to reduce relative deprivation, people should start from selecting appropriate comparison objects. The public should be guided to choose suitable comparison objects and make a reasonable comparison.

### The Comparison of Relative Deprivation of University Students With Other Social Groups

The survey shows that the current university students have a high level of relative deprivation. There are similar findings for other groups. Guo (2001) found in a survey that urban residents had a high level of relative deprivation, and a group with low social status and income presented the highest level of relative deprivation. Wang (2007) also found that the same truth applied to urban retirees. The high relative deprivation level of university students can be explained by large disparity among these students, such as family income, family background, socioeconomic status, academic performance, and a relationship, all of which become an aspect of social comparison and cognitive evaluation. The high relative deprivation level has a negative impact on the mental and physical health of students. Zhang and Tao (2013) conducted studies on students of a university in Beijing and found that the high relative deprivation level had a significant positive correlation with suicidal inclination and depression, and a significant negative correlation with social support. Therefore, it is necessary to pay attention to their mental and physical health and reduce their relative deprivation. The relative deprivation scale designed in this study for university students can effectively measure the relative deprivation of university students, which helps understand their mental condition, so that intervention measures can be taken in a timely manner to reduce negative mental process.

In the demography factor difference analysis, the research found that there is no significant difference in relative deprivation in gender, but with the grade, there exists a significant difference. And the relative derivation has increased along with the grade raise. The research suppose that the situation of relative deprivation of university students may be the same of campus life, such as the schoolwork, friendship, date and love, examination, strive to get certification, further study, job hunting, and daily file. Whether female or male students, they may need to resolve the similar questions at all. Therefore, the research found a non-significant difference in relative deprivation. Regarding grade factors, the students may confront difficult tasks and problems when they get to grow. When they get a higher grade year by year may face the following problems: tougher academic tasks, the more competition to get progress, the tension in daily life, the difficulty to make choice, and the with those predicament and challenge exacerbate, the students had confront increased stress, but the coping capacity was have not promoted synchronized, the students may turn into differentiation, varied to be well-adapted or maladjustment. The complicated variation of students has caused the social comparison, cognitive evaluation, and emotional feeling to be changed, so the relative deprivation was increased with the grade.

### Summary

After the open questionnaire survey was conducted and the opinions of psychologists and doctors in social psychology were solicited, the items for the questionnaire were finally determined. When designing the questionnaire, the description, details, semantics, and other aspects of the items were evaluated repeatedly in order to ensure that the questionnaire had high content validity. The reliability test indicates that the relative deprivation questionnaire and every item has high internal consistency reliability and retest reliability, all of which meet the criteria of psychometrics. In summary, all indicators, such as reliability and validity, of the USRDQ meet the requirements of psychometrics and can be used to measure the relative deprivation of university students. Only when the relative deprivation is measured effectively, it can interpret the comparison of people upward.

## Future Research

The USRDQ designed in this study measured relative deprivation resulted from social comparisons with reference objects. As respondents in this study are all university students, the validity of the questionnaire has not been verified for other groups, which may limit the application of the questionnaire. However, the questionnaire contains the main contents of social comparison of relative deprivation, so it can also be applied to other groups. Based on this questionnaire, future studies can focus on designing a new relative deprivation questionnaire for other groups (e.g., socially vulnerable groups) or social members to see whether structural dimensions of relative deprivation should be adjusted, and test the reliability and validity of the questionnaire.

## Conclusion

The USRDQ designed in this study has high validity and reliability. USRDQ targets the university students and is more accurate in measuring relative deprivation than other scales. The research proved the relative deprivation theory of Smith et al. (2012). Relative deprivation has three dimensions, namely, social comparison, cognitive evaluation, and emotional experience.

## Data Availability Statement

The original contributions presented in the study are included in the article/supplementary material, further inquiries can be directed to the corresponding author/s.

## Ethics Statement

Ethical review and approval was not required for the study on human participants in accordance with the local legislation and institutional requirements. Written informed consent for participation was not required for this study in accordance with the national legislation and the institutional requirements. Written informed consent was obtained from the individual(s) for the publication of any potentially identifiable images or data included in this article.

## Author Contributions

LJ contributed to data analyses, writing of the manuscript, critical review of the manuscript, and formal analysis.

## Conflict of Interest

The author declares that the research was conducted in the absence of any commercial or financial relationships that could be construed as a potential conflict of interest.

## Publisher’s Note

All claims expressed in this article are solely those of the authors and do not necessarily represent those of their affiliated organizations, or those of the publisher, the editors and the reviewers. Any product that may be evaluated in this article, or claim that may be made by its manufacturer, is not guaranteed or endorsed by the publisher.
